# Effect of CRP on Some of the in vitro Physicochemical Properties of LDL

**Published:** 2010

**Authors:** Hashem Nayeri, Gholam Ali Naderi, Masoud Saleh Moghadam, Samaneh Mohamadzadeh, Maryam Boshtam, Narges Jafari Dinani, Adel Abedpour Dehkordi

**Affiliations:** 1 Assistant Professor of Biochemistry, Islamic Azad University, Falavarjan Branch, Department of Biochemistry, Falavarjan, Iran; 2 Associate Professor of Biochemistry, Isfahan Cardiovascular Research Center, University of Medical Sciences, Isfahan, Iran; 3 Associate Professor of Biochemistry, Department of Biochemistry, University of Payame Noor (Pnu), Mashhad, Iran; 4 MSc, Animal Physiology, Isfahan Cardiovascular Research Center, Isfahan University of Medical Sciences, Isfahan, Iran; 5 MSc, Animal Physiology, Isfahan Cardiovascular Research Center, Isfahan University of Medical Sciences, Isfahan, Iran; 6 MSc, Animal Physiology, Islamic Azad University, Falavarjan Branch, Department of Biochemistry, Falavarjan, Iran; 7 MSc, Student of Biochemistry, Department of Biochemistry, Payame Noor University (Pnu), Mashhad, Iran

**Keywords:** Atherosclerosis, C reactive protein, Low-density lipoprotein, Inflammation

## Abstract

**BACKGROUND:**

Atherosclerosis is the most important underlying cause of cardiovascular diseases (CVD) which recently has been classified as an inflammatory disorder. Accumulation of large amounts of oxidized LDL in the intima during local inflammation reaction led to increase several factors such as C -reactive protein (CRP). It has also been reported that CRP is able to bind with modified forms of LDL as well as oxidized LDL. These findings suggest possible positive or negative involvement of this protein in atherogenesis. The main objective of the present study was to assess the influence of CRP on LDL oxidation and the possible physical \changes of LDL in the presence of CRP in vitro.

**METHODS:**

In this study, the susceptibility of purified LDL to oxidation was assayed by monitoring of formation of conjugated dienes in different physiological concentrations of CRP (0 - 0.5 -2 µg/ml) using a shimadzu spectrophotometer. Electrophoresis was used to determine the electrophoretic mobility of LDL in those conditions.

**RESULTS:**

CRP significantly reduced the susceptibility of Cu^++^ -induced LDL oxidation through increasing the lag timeand there was positive relationship between these findings and CRP concentration (P < 0.05). CRP caused a significant reduction in the electrophotretic mobility of LDL compared to native LDL (n-LDL) (P < 0.05).

**CONCLUSION:**

A considerable reduction was shown in LDL oxidation, in higher concentration of CRP, via an unknown mechanism. The electrophoretic mobility of LDL, in the oxidative condition, decreases in the presence of CRP compared to n-LDL, which can be indicative of the effect of this protein on the physical and chemical properties of LDL. It seems that, other pathway than LDL oxidation is responsible for the effect of CRP on the atherogenesis processes.

## Introduction

Coronary Artery Disease (CAD) is one of the most common causes of death worldwide and atherosclerosis has been identified as its main underlying cause.^1^ Atherosclerosis is the formation of fibrous lesions and fatty streaks in vessels, which is associated with inflammation.^2^ Thus far, researchers studying various factors suspected of triggering this pathological process have achieved valuable results, however, as yet no specific pathogenic process has been identified as the initiating point in the process of atherogenesis in vessels and all our information consider it to have a multifactorial nature caused by an interplay of linked pathological mechanisms.^3^ Those responsible for LDL oxidation consider as the most important pathological processes. Ox-LDL is the end product of LDL oxidation in the blood, which is highly destructive atherogenic. Ox-LDL is one of the principal triggers of inflammatory reactions in vascular endothelial cells which stimulate the secretion of cellular inflammatory markers.^4^ During the past years, extended studies have been conducted to identify the factors involved in oxidation and modification of lipoproteins, especially LDL. Some of these studies have focused on inflammatory markers.^1^–^5^ Level of these markers increase during inflammatory process and it is suggested that they may be involved in atherogenesis.^6^

In some studies, the interaction of some of these markers and LDL has been reported, including in a study which reported CRP binding with modified forms of LDL; it was even observed that it binds with native LDL .^7^ Nunomura et al. in 1990 demonstrated that this interaction probably occurs via apolipoprotein B in LDL at phosphorylcholine binding site of CRP.^8^ The mechanism activated by CRP through this binding and its nature (positive or negative) remains a matter of debate. The findings of previous studies in this field are controversial.^9^–^12^ Therefore, this study attempted to address one of the challengeable aspects of CRP. It was assumed that CRP influences LDL oxidation after binding with it and affects some of its physical properties. Our study showed that CRP can induce various properties in relation with LDL. For example, it was observed that it reduced the susceptibility of LDL to oxidation and decreased the electrophoretic mobility of oxidized LDL. In general, finding the relationship between these effects and the atheorgensis process warrants further studies in this field.

## Materials and Methods

1. ***Blood sample collection*****;** In the beginning, a 100 ml sample of a healthy man's blood was prepared and centrifuged at 3000 rpm for 15 minutes.
2. ***LDL purification*****;** LDL was extractedseparated from the fresh serum using a Beckman coulter optima L-100XP ultracentrifuge, with a two-stage ultracentrifugation technique described by Bronzert & Brewer in the specific concentration gradients at 60000 rpm for 6 and 12 hours respectively, at 16 °^C^ [13].
3. ***LDL dialysis*****;** The obtained LDL was carefully poured into a Sigma-Aldrich® dialysis tube and after closing both ends of the tube, it placed in a dish containing PBS buffer (i.e. 16.7 mM sodium dihydrogen phosphate dihydrate and 21.1 mM sodium phosphate dibasic in 160 mM sodium chloride with a pH of 7.4) for 24 hours at 6 °^C^ on an electric shaker in a dark room.
4. ***Determining proteinconcentration (Lowry method);*** To conduct the later stages of the study, the concentration of LDL protein was measured using Lowry's standard method.^14^
5. ***Oxidation of LDL*****;** The susceptibility of LDL to oxidation was detected using a standard spectrophotometric method. Shimadzu spectrophotometer UV 3100 was used to monitor formation of conjugated dienes based on the technique proposed by Esterbauer and his colleagues at the wavelength of 234 nm.^15^ At first, control sample containing 20 µg-protein/ml of LDL and 5 µM copper sulphate in PBS buffer, pH=7.4 in a quartz cuvette was monitored, then test samples which in addition to contents mentioned in the control sample contained 0.5 µg/ml and 2 µg/ml of CRP were assessed with the same technique respectively (All the experiments were repeated three times, data were analyzed using student's *t*-test by PRISM^©^ 5).
6. ***Electrophoresis*****;** Electrophoretic mobility of normal LDL (∼10 µg protein in a 10 µl sample) compared to LDL incubated with CRP (∼2.5 µg/ml), also ox-LDL which obtained by incubating of LDL (∼10 µg protein in 10 µl sample) with 5 µM copper sulphate compared to ox-LDL incubated with CRP (∼2.5 µg/ml) in 1% agarose gel (AGE). Electrophoresis was performed for 150 min at 90 V in 0.05 M barbital buffer, then migrated LDLs were fixed in the gel with methanol: water: glacial acetic acid (6:3:1) and finally stained by 0.1% fatRed-7B in methanol, which just before use should be mixed with 0.1 N NaOH.

## Results

**Figure 1:**
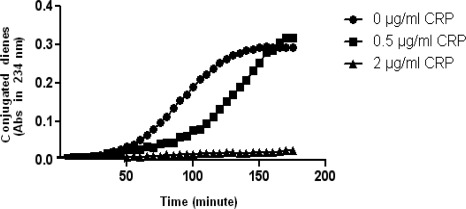
Inhibition of LDL oxidation is proportionate with increased CRP concentration. The curves represent the susceptibility of LDL to oxidation at the concentration of 20 µg protein/ml, in presence of different physiologic concentrations of CRP. In 0.5 µg/ml of CRP (•), susceptibility of LDL to oxidation (copper sulphate concentration: 5µM) relatively reduced through increasing the lag time, and when CRP concentration reached 2 µg/ml (▲), the oxidation of LDL was inhibited in the time frame (Data points are mean of three measurements. Significance between lag times were analyzed using student's t-test and were significant (P < 0.05). In table 1, various quantative kinetic parameters, associated with steps of oxidation of LDL in the presence and absence of CRP have shown. The data which obtained from the findings of figure 1 show; lag time, maximal rate of oxidation, time need to reach maximal amount of dienes and maximal amount of dienes formed. In lower concentration of CRP, the differences were significant only in lag time, but in higher concentration of CRP, because of sizable inhibition of oxidation, only maximal rate was detectable in the time frame and its difference was significant, other parameters were not detectable.

Initially, CRP in the concentration of 0.5 µg/ml reduced the susceptibility of LDL (20 µg-protein/ml) to oxidation through increasing the lag time and when its concentration reached 2 µg/ml, it nearly inhibited LDL oxidation in the defined time (Figure 1). Other oxidation parameters such as maximal amount of dienes formed (diene (max)), maximal rate of oxidation (maximal rate) and time need to reach maximal amount of dienes (t (max)) in CRP (0.5 µg/ml)-incubated LDL samples compared to equal parameters in alone LDL samples, had no significant statistical differences (P > 0/05). On the other hand, maximal rate of oxidation in CRP (2 µg/ml) -incubated LDL samples, had significant difference with LDL samples without CRP (P < 0.05). Other oxidation parameters in the latter concentration of CRP were not detectable in the time frame (Figure 1 and Table 1).

**Table 1:** Quantative parameters of oxidation of LDL

CRP (µg/ml)	Lag Time (min)	Maximal Rate (µM/min)	t (max) (min)	Diene (max) (A234 nm)
0	55 ± 5	0.115 ± 0.009	155 ± 5	0.3 ± 0.01
0.5	95 ± 7 *	0.136 ± 0.011	170 ± 8	0.28 ± 0.02
2	Unknown in the time frame	0.008 ± 0.005 *	Unknown in the time frame	Unknown in the time frame

Values are shown as mean ± SD

* Significantly different from mean values of samples which contain 0 µg/ml CRP, according to student's t-test(P < 0.05).

**Figure 2:**
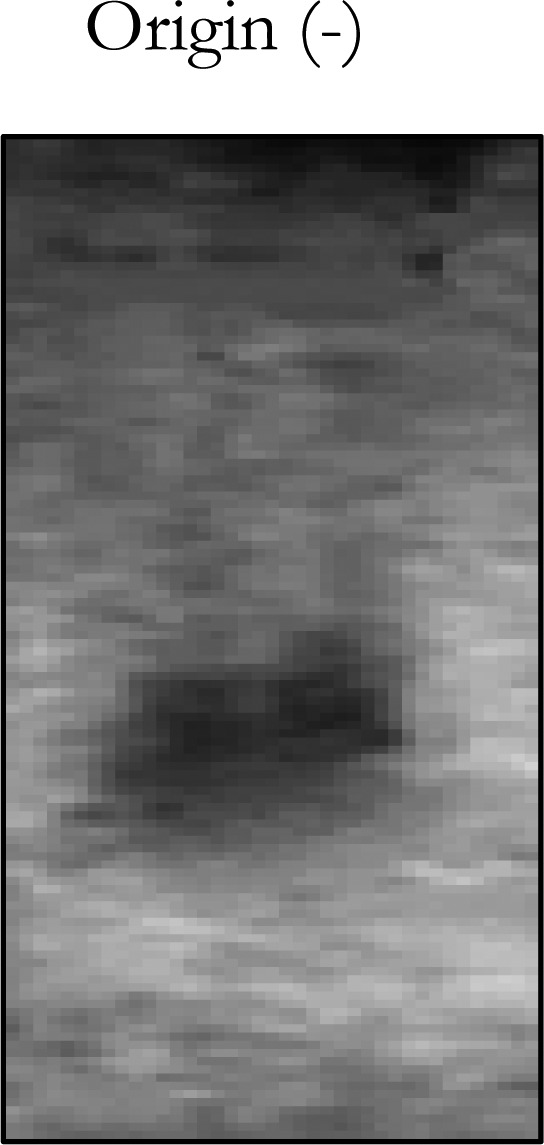
Electrophoresis of isolated LDL is indicator of purity of LDL (∼10 µg protein in a 10 µl sample). It performed in 1% Agarose gel at 90 volts during 150 minute, then staining process performed with FatRed 7B.

In figure 2, the single LDL bond which was separated using ultracentrifuging method is shown. The single bond indicates the purity of separated LDL, without any contamination with other lipoproteins.

**Figure 3:**
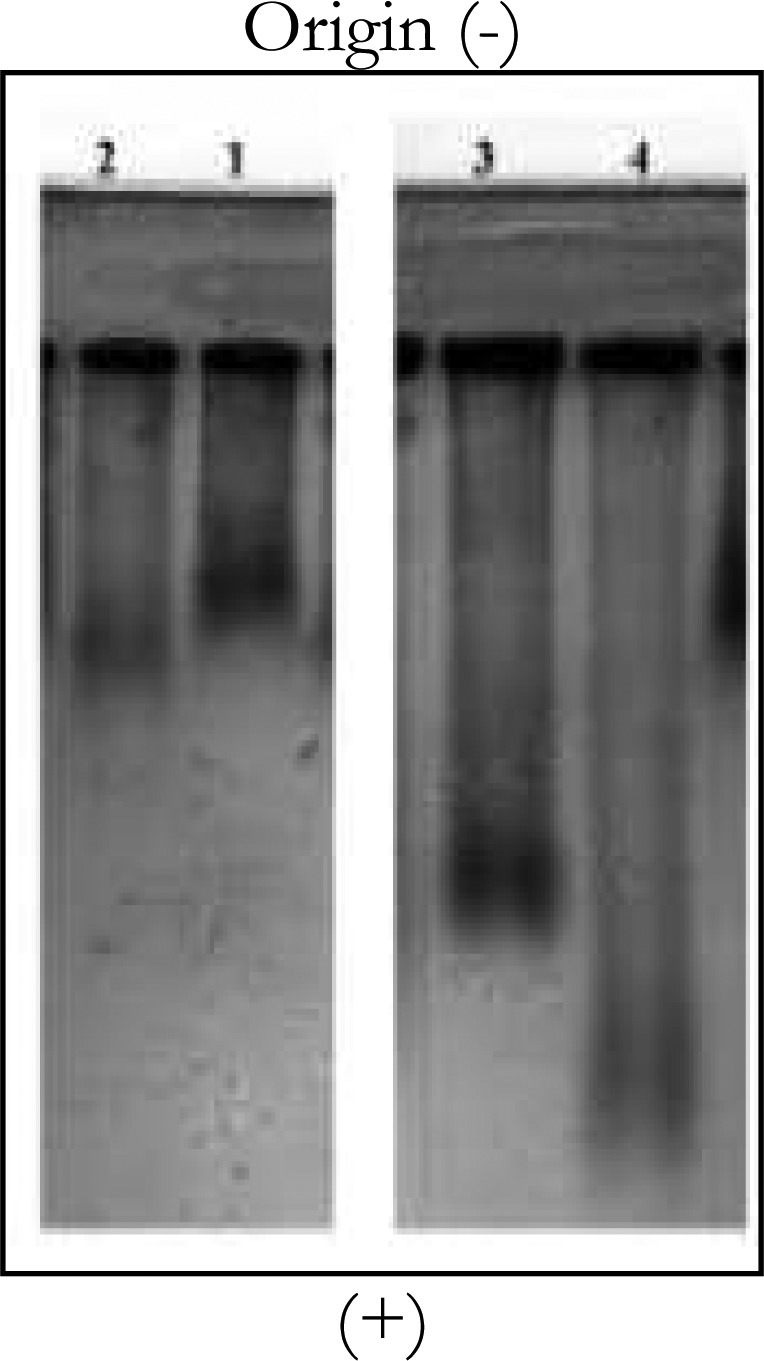
Electrophoresis of incubated samples of LDL and CRP in Agarose gel

According to Figure 3, the LDL (∼10 µg protein in a 10 µl sample) which was kept in the presence of CRP for 18 hours (number1) only had a relatively reducecd electrophoretic mobility (reduced REM) compared to LDL alone (number 2). LDL which was incubated in the presence of CRP and oxidative factor, i.e., 5µM Cuso4 for 18 hours (number 3) showed significant reduction in electrophoretic mobility (considerable reduced REM) compared to LDL which only incubated with copper ion in the same incubation time (number 4), which can be due to reducing of negative charge on the surface of ox-LDL particle in the presence of CRP.

## Discussion

Pentameric acute phase reactant protein or CRP is a sensitive risk marker of cardiovascular diseases, as confirmed by recent studies .^17^, ^18^ However, the possible role of CRP in atherogenesis as a risk factor or even a useful factor with antiatherogenic properties is still questionable which can be seen in the occasionally contradictory results of previous studies .^9^–^12^, ^19^ The present study shows that CRP is able to inhibit the invitro oxidation of LDL in a direct relationship with concentration, Nonetheless the biochemical mechanism whereby this protein shows this ability is not clear. The result of electrophoresis in this study shows that the LDL which was in the presence of the oxidant copper cation and CRP, had less electrophoretic mobility than LDL which was only in the presence of copper cation. This may be due to the fact that CRP has conferred new physicochemical properties on semi-oxidized LDL by binding to it and decreases its negative surface charge and consequently reduces its electrophoretic mobility. This latter conclusion is consistent with the recent findings of Zwaka et al. and Paceri et al. that CRP can facilitate the phagocytosis of semi-oxidized LDL through macrophages which ultimately leads to formation of atherogenic foam cells.^9^, ^11^–^13^ The results of this study which are indicative of relative reducing in negative surface charge of LDL particle in the presence of CRP, are in line with the results of a study conducted by Rufail et al. It is noteworthy that in that study, the result of electrophoresis was obtained only after 2 hours of incubation, but in the present study, this period was extended to 18 hours.^19^ It can be explained that possibly this protein participates in atherogenesis through a mechanism leading to conformational changes in the semi-oxidized LDL molecule.

The results of this study are consistent with recent studies which have highlighted a kind of relationship and physical binding between CRP and Ox-LDL.^7^, ^8^, ^20^ It is possible that binding of CRP to some areas of the LDL particle (like, phosphocholine moiety) leads to some changes on the surface of this lipoprotein, resulting in reducing of susceptibility of LDL to more oxidation in addition to preparing this modified-lipoprotein for phagocytosis by macrophage receptors.

In the present study, different level of CRP within its physiological range in serum were used to assess the degree to which LDL oxidation would be influenced in progressive concentrations (0 µg/ml, 0.5 µg/ml and 2 µg/ml CRP) because according to previous studies, it has been clearly demonstrated that the elevated level of CRP is related to higher incidence of cardiovascular disease (17, 18, 22). It is notable that the results of some recent studies have shown that vascular endothelial cells are prompted by a number of trigger factors to secrete CRP^22^ this finding can form a basis for the hypothesis that this protein may be dispatched to the external space, where LDL is invaded by oxidative factors. Also one could hypothesize that LDL, which in the initial stages of oxidation induces CRP secretion, indirectly recalling this protein towards itself to resist invading factors. Based on the result of this study, maybe the processes hypothetically ascribed to CRP be inherently conducive to vascular health and this protein may have antiatherogenic properties by providing relative protection for LDL against oxidation, but it must be borne in mind that it may be possible that, under acute conditions,it act to expedite atherogenesis (such as; cooperation with macrophages to form foam cells). This second hypothesis may be more compatible with the fact that the level of CRP increases during cardiovascular diseases.^9^, ^11^, ^12^, ^19^

## Conclusion

Overall, the effect of CRP on the atherogenesis processes may pass through a pathway other than LDL oxidation,indepenently from the mechanism whereby LDL oxidation is inhibited. More comprehensive studies should be done to determine whether CRP has a truly conspicuous role in atherogenesis and inflammatory abnormalities or not.
